# Comparison of the Measurement of Long-Term Care Costs between China and Other Countries: A Systematic Review of the Last Decade

**DOI:** 10.3390/healthcare8020117

**Published:** 2020-04-29

**Authors:** Qingjun Zeng, Qingqing Wang, Lu Zhang, Xiaocang Xu

**Affiliations:** 1 School of Economics, Chongqing Technology and Business University, Chongqing 400067, China; zqj@ctbu.edu.cn (Q.Z.); melodyzl@163.com (L.Z.); 2 Research Center for Economy of Upper Reaches of the Yangtse River, Chongqing Technology and Business University, Chongqing 400067, China; wqq199412@163.com; 3 Department of Actuarial Studies & Business Analytics, Macquarie University, Sydney 2109, Australia

**Keywords:** Long-Term Care (LTC), long-term care costs, disabled elderly, institutional care, home care

## Abstract

Background: The rapid aging of populations in some countries has led to a growing number of the disabled elderly, creating a huge need for Long-Term Care (LTC) and meeting its costs, which is a heavy economic burden on the families of the disabled elderly and governments. Therefore, the measurement of Long-Term Care (LTC) costs has become an important basis for the government to formulate Long-Term Care (LTC) policies, and academic research on Long-Term Care (LTC) costs is also in the process of continuous development and deepening. Methods: This is a systematic review that aims to examine the evidence published in the last decade (2010–2019) regarding the comparison of the measurement of Long-Term Care (LTC) costs between China and other countries. Results: Eighteen Chinese studies and 17 other countries’ studies were included in this review. Most Chinese scholars estimated long-term care costs based on the degree of disability among the disabled elderly. However, the studies of European and American countries are more and more in-depth and comprehensive, and more detailed regarding the post-care cost of specific diseases, such as Parkinson’s disease, Alzheimer’s disease, and epilepsy. Conclusion: In future academic research, we should fully consider the human value of long-term care providers and further study the differences in the long-term care costs of different chronic diseases. In China’s future policymaking, according to the experience of Germany, Sweden, and other countries, it may be an effective way to develop private long-term care insurance and realize the effective complementarity between private long-term care insurance and public long-term care insurance (LTCI).

## 1. Introduction

The increasingly aging population has highlighted the urgency of the crisis in healthcare services for the elderly in China in recent years. According to World Population Prospects (2019), published by the Department of Economic and Social Affairs of United Nations (WPP2019), population aging is expected to increase rapidly (South Korea, 38.1%; Japan, 37.7%; Italy, 36.0%; Germany, 30.0%; and China, 26.1%) by 2050 [1]. According to CSY (China Statistical Yearbook, 2019), the elderly population, aged 65 or above, reached 167 million in 2018, accounting for 11.9 percent of the total population. The ODR (old-age dependency ratio) climbed from 9.9% in 2000 to 16.8% in 2018 [2]. The average life expectancy in China is expected to reach 81.52 years in 2045–2050, which is close to the average of 83.43 years in developed countries (WPP2019). Among them, the number of disabled elderly due to chronic diseases [3], industrial or agricultural environmental pollution [4,5,6,7], accidental injuries, and natural aging is increasing sharply. Accordingly, the demand for LTC (Long-Term Care) services and its cost for the elderly are growing rapidly [8,9]. However, the provision of LTC (Long-Term Care) services such as policy formulation, operational models, and, especially, fund-raising lag far behind in China. As a result, the “Healthy China Strategy” has been proposed in the Report of the 19th National Congress of China.

Long-Term Care (LTC) refers to the various supportive personal and social services needed by people who are unable to take care of themselves for a long time. The existing definitions differ in the causes of incapacitation and the content of services, leading to the definition of LTC being somewhat vague. For instance, the World Health Organization (WHO) considers LTC to be “a system of care activities carried out by informal caregivers (family, friends or neighbors) and professionals (health and social services) to ensure that those who do not have full self-care capacity continue to enjoy a higher quality of life” [10]. The National Institute on Aging (NIA) indicates that “Long-term care involves a variety of services designed to meet a person’s health or personal care needs during a short or long period of time. These services help people live as independently and safely as possible when they can no longer perform everyday activities on their own” [11]. The Health Insurance Association of America (HIAA) defines long-term care more broadly, stating that Long-Term Care (LTC) refers to “the care provided to people with chronic diseases, cognitive impairment, such as Alzheimer’s disease or in a disabled state, that is, functional impairment, over a long period, which is made up of medical services, social services, home services, delivery services, or other support services” [12]. The difference between the HIAA’s and NIA’s definitions of “Long-Term Care (LTC)” is mainly reflected in three aspects. Firstly, the causes of service demand can include physical impairment and cognitive impairment. Secondly, the categories of services include both services involving the basic needs of the elderly, such as personal care and daily living, and extended and supportive services, such as healthcare and psychological care. Thirdly, the long-term care service forms can be either home nursing, that is, professional nursing personnel door-to-door service, or institutional nursing, that is—including in nursing homes—apartments for the elderly and other non-hospital professional nursing institutions for professional care. The definition of LTC by the HIAA has been widely used in academic discussions and the construction of the long-term care service system in China. Therefore, this paper makes some special definitions regarding LTC. Firstly, the target group of LTC service is only defined as the elderly, although children are not excluded from the definition of LTC. Secondly, the service categories of LTC include both daily care and healthcare after hospital care. Therefore, the differences between the postoperative care costs of different diseases can also constitute a discussion topic regarding the cost of LTC. Thirdly, the care forms of LTC consist of institutional care, community care, and home care. Even in recent years, some countries, such as China, have tried many innovations for the continuous improvement of long-term care service systems. For example, despite the name of “hospital”, a “hospital for the elderly” is essentially a nursing institution similar in nature to nursing homes.

Long-term care systems have made great progress in developed countries since the 1960s. The LTC system in the UK is less dependent on public funds than in most other European countries. Its LTC services are financed by the National Health Service (NHS) and local governments [13]. In Japan, the Long-Term Care Insurance (LTCI) system was implemented in 2000 to cope with the growing costs of LTC. The applicant can decide the care level according to the amount available, and care beyond the fixed amount must be undertaken privately [14,15,16]. Sweden established a tax-based LTC system in the 1970s. Every city must provide help if the care demands of an individual cannot be satisfied [17]. In general, the scope of LTC and ability to meet the cost of providing it have been limited since a severe economic crisis in the 1990s. As a consequence, those countries will face substantial sustainability problems in providing LTC in the future. In China, the demand for LTC services has increased dramatically due to the aging population. The number of people aged 65 and above increased from 96 million in 2003 to about 150 million in 2018 [18]. However, the supply of LTC services, such as fund-raising, lags far behind. However, the government have introduced a series of policies and measures, such as the pilot of LTC insurance (LTCI), to establish a comprehensive LTC service system, in recent years.

The major difference between the U.S. and the other countries was the level of public funding, which was universally provided in Sweden, Denmark, and the Netherlands but only provided publicly by the U.S. to the disabled and indigent population or in a limited way to individuals after a hospital stay. The level of public funding is nearly 100% for Denmark, Sweden, and the Netherlands but only 72.9% in the United States [19]. As a result, the total U.S. long-term care spending paid out of pocket by patients and/or families in 2011 was $45.5 billion [20]. Cash for care programs have been reported to be a part of all three European countries’ LTC publicly provided programs, but this was not the case in U.S., which does not yet have a national model for these programs. Additionally, the U.S. LTC system lacked coverage for a large number of citizens compared to the systems of other OECD countries, where the major flaws of their LTC systems were the excessive costs and poor sustainability.

Compared with the systems in European and American countries, China’s Long-Term Care (LTC) system started late and developed relatively slowly. For example, China’s government-led public Long-Term Care Insurance (LTCI) has only been officially piloted in 15 cities, including Qingdao, Chongqing, and Shanghai, since 2016. Therefore, it is very important for China to learn from the valuable experience of Germany, Sweden, and other European and American countries that started to implement the Long-Term Care (LTC) system in the 1990s, as well as their relevant theoretical research. This review aims to examine the evidence published in the last decade (2010–2019) regarding the measurement of Long-Term Care (LTC) costs between China and other countries, and through the comparisons, to explore the academic space for further research, with a hope to provide policy recommendations for the construction of China’s Long-Term Care (LTC) system.

## 2. Materials and Methods

This review was carried out to explore related studies published in the last decade (2010–2019) regarding the comparison of the measurement of Long-Term Care (LTC) costs between China and other countries.

### 2.1. Literature Search Source

Our review was carried out to show the measurement of Long-Term Care (LTC) costs in related studies. The databases mentioned in our review mainly were: Web of Science, Medline, SCOPUS, EBSCO, PubMed, and CNKI (China). The following combinations of terms were used, with the Boolean phrase "and/or", to maximize the scope and type of material referred in the search: “Long-Term Care cost” OR “LTC cost”. The search was carried in Chinese and English. We also added a Chinese research database named VIP (China) to look for any studies that might be missing.

### 2.2. Data Extraction Criteria

Preliminary inclusion criteria: the selection criteria for publications were as follows: (1) studies on the measurement of Long-Term Care (LTC) costs; (2) research conducted in China and other countries, which should be strictly separated to facilitate subsequent analysis; and (3) research published from January 2010 to December 2019. Publications based on opinions or comments, editorials, and summaries of meetings were not included.

Exclusion criteria: these included articles reporting on the results of a qualitative study, quantitative analyses, surveys, feasibility studies, relationship measurements, satisfaction studies, “what-if” analyses, and data collection techniques. Publications based on opinions or comments, editorials, and summaries of meetings were not included. The results were initially extracted by one researcher and then cross-checked by another to ensure that all data had been screened and reviewed. If there was a difference of opinion between the two researchers, a third researcher was invited to express their opinion and finally reach an agreement. All publication information was exported to the Excel database via Endnote, and duplicate sections were removed. We followed some previous special definitions regarding LTC as described previously in this paper. Finally, 18 Chinese studies and 17 other countries’ studies were retained for the review.

**Figure 1:**
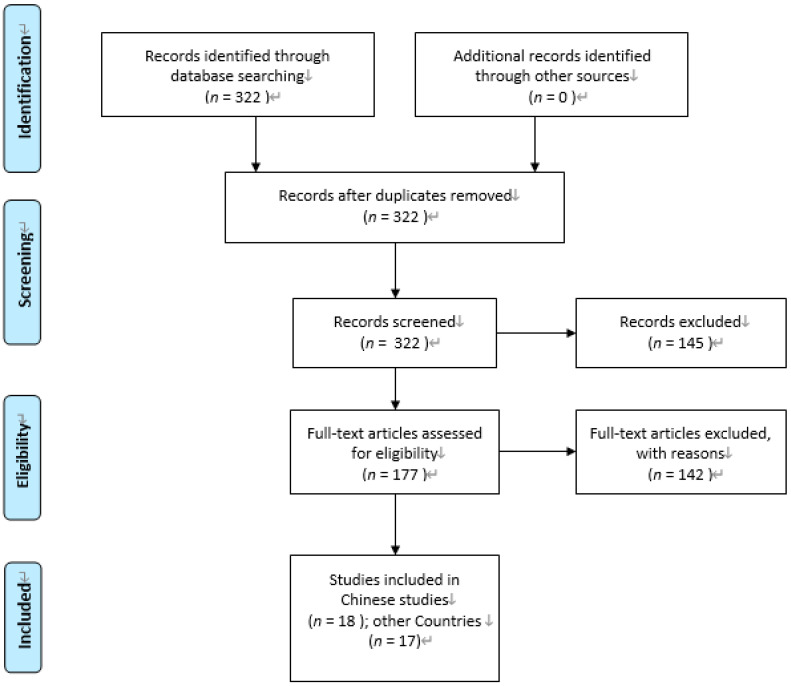
Flow diagram of study selection (PRISMA). Note: Moher D, Liberati A, Tetzlaff J, Altman DG, The PRISMA Group (2009) [21].

The information extracted from all the included publications was as follows: time frames, study methodology (design, purpose, participants, and tools), samples, and key conclusions. All the analysis results were analyzed in the following process (as shown in Figure 1).

### 2.3. Quality Assessment

Study quality was independently assessed by the two researchers using the Risk of Bias Assessment Tool for Non-Randomized Studies (RoBANS) [22]. The criteria included the selection of participants, confounding variables, intervention measurements, the blinding of outcome assessments, the incompleteness of outcome data, and selective outcome reporting. Each criterion was evaluated as “low risk of bias”, “high risk of bias”, or “unclear”. In cases of disagreement, each case was discussed with a third researcher. The quality of the included studies is summarized in Table 1.

## 3. Results

### 3.1. A Preliminary Review of the Relevant Literature

It was found that three-quarters of the studies were made up of quantitative research, while qualitative research only accounted for one quarter. Moreover, the qualitative method was used in most research in China, while quantitative methods could be found in nearly every article in other countries, in which the acceptance by and understanding of the readers could be better.

From the publication point of view, the journals that published the most related papers were *BMC Health Services Research*, *Geriatrics & Gerontology International*, *PLOS One*, *Public Health Nursing*, *Nursing Research* (Chinese), and *Chinese Health Economics* (Chinese), respectively. Among them, *BMC Health Service Research* and *PLOS One* were tied at 15% each, and *Geriatrics & Gerontology International* and *Public Health Nursing* each had 10%. Besides, it was interesting that some of them were nursing and health, biology, physics, and other comprehensive journals, which are not pure medical journals. 

#### 3.1.1. Classification by Country Studied

**Figure 2:**
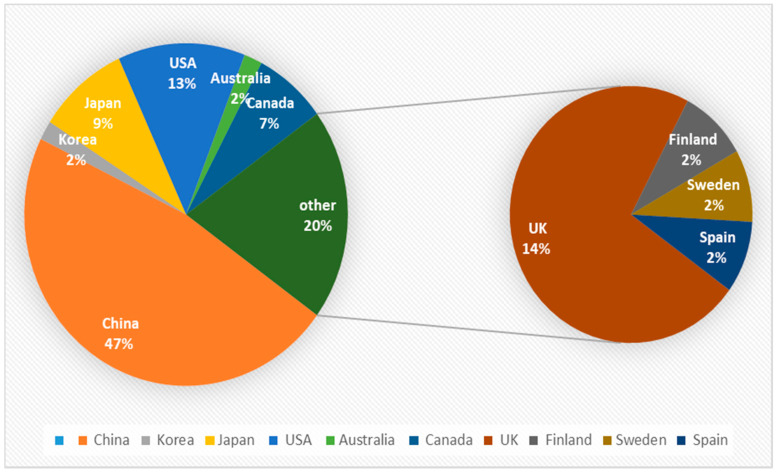
Classification of publication by country studied.

The classification of articles published regarding the measurement of LTC (Long-Term Care) costs in different countries is presented in Figure 2. 

It can be seen from Figure 2 that the pie chart on the left is divided into eight parts according to the research object for LTC in related articles. It was clear that China has become the most popular research object, accounting for nearly half of all the published articles, and the study of LTC in the UK ranked the second. Meanwhile, the United States came in third place, accounting for 14% of the pie chart. It can be observed that there is a small circle on the right, composed of Spain, Sweden, Germany, and Finland. 

#### 3.1.2. Classification by Publication Year

**Figure 3:**
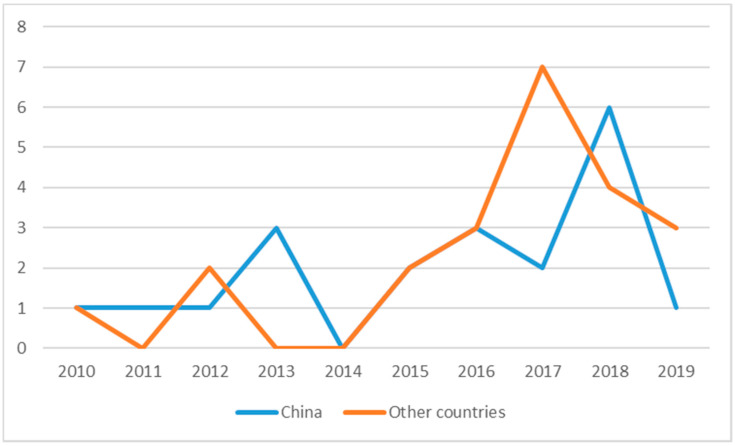
Classification of publication by year.

The classification of articles published on LTC (Long-Term Care) costs across the timeline of the study (2010–2019) is presented in Figure 3. 

It can be seen from Figure 3 that the amount of research on LTC is not large in general and currently in a declining state, with peaks in 2017 and 2018. Nevertheless, it has shown a wavy upward trend. The attention of some scholars had been attracted to LTC since 2010. After a brief decline in 2014, it peaked in 2017 and 2018. Based on the analysis above, it can be inferred that more and more scholars at home and abroad have devoted themselves to the research of long-term care costs since 2014 and gradually achieved excellent results. The result could be attributed to the increasing emphasis on nursing in various countries. At the same time, it was reflected that the development of social structures in various countries is becoming more and more perfect.

### 3.2. Measurement of Long-Term Care Costs in China

In 2010, the cost accounting of home care programs was shown by Jin et al. [23], and the research into long-term care costs is getting more and more diversified (as shown in Table 2).

Most Chinese scholars estimated long-term care costs based on the degree of disability among the disabled elderly. For example, Lu [24], Feng et al. [25], Hu [26], and Yuan [27] calculated the basic LTC costs based on the Activities of Daily Life (ADL) classification of mild, moderate, and severe dysfunction. In addition, Liu and Zhong [28] compared the LTC costs generated by patients with different ADL function obstacles with the living allowances in urban and rural areas, which could be a new reference for the long-term care cost system. Xu et al. used the Bayesian quantile regression method to measure the high, medium, and low levels of long-term care cost prediction for each disability state of the elderly in China from 2020 to 2050 [29].

Other Chinese scholars focus on how to integrate the human value of nursing staff into the measurement system for long-term care costs, so as to achieve the goal of the scientific and reasonable measurement of long-term care costs. The value of nurses was noticed in the studies of Fengyue and Junko [30], Yu et al. [31], and Fan et al. [32], which discussed the LTC costs in Chongqing, Guangdong, and other provinces in China. Qun [33] compared the LTC costs in nine provinces of China with those in Texas, U.S. Li et al. constructed a pricing model for nursing service projects that can measure the value of LTC services and fully reflect the LTC costs [34]. With the development of the nursing industry, a variety of nursing services could be offered by nursing institutions, communities, and even in homes, aside from hospitals. The process of providing home care services in a community of Shanghai was discussed by Du et al. [35]. They calculated the cost of five plates, including manpower, materials, and another three big plates, and it was found that the current charge for the home care services could not cover the necessary expenditures. 

The comparison of long-term care costs under different forms of care (such as institutional care, community care, and home care) has become another important topic of academic discussion regarding the development of elderly care institutions in China. Yang et al. [36] and Lu et al. [37] revealed that the total cost of institutional care is far less than the cost of general hospital inspections and other routine expenses. The direct and indirect costs of home care were calculated by Huang et al. [38] and Song et al. [39]. It can be concluded from their research that institutional care is suitable for patients whose daily life is marked by high dependence, while home or community care could be a better option for the less dependent. Then, a survey of the influencing factors for the utilization and cost of formal care in the elderly community of Shanghai was conducted by Fen et al. [40], who found that professional home care was more cost-effective compared with the care provided by family members.

In a word, LTC-related research is a relatively new concept in China. With the pilot practice of the LTCI system in Qingdao, Shanghai, Chongqing and other places, LTC-related research has begun to rise. However, due to the lack of official statistics and uniform standards of LTC costs, these studies have many difficulties, especially in empirical studies.

### 3.3. Measurement of Long-Term Care Costs in Other Countries

Developed countries such as those in Europe and the United States have paid attention to long-term care costs earlier, and the research objects were more diversified and comprehensive (as shown in Table 3). It was found by Ryan Greysen [41] that the LTC costs for the elderly with the highest level of functional disorders were 77% higher than for those without functional disorders. Lagergren et al. [42] predicted the future LTC costs in Japan and Sweden based on age, gender, and degree of ADL dysfunction. 

As the research on long-term care is more and more in-depth and comprehensive, the research on long-term care costs was more detailed regarding the post-care costs of specific diseases. Some studies on the LTC costs of Parkinson’s disease [43,44,45], Alzheimer’s disease [46,47,48], colorectal cancer [49], and viral gastroenteritis [50] were conducted. In addition to cancer, which has been a problem for a long time, even research on the LTC costs of alcoholism was carried out by Edward et al. [51]. Overweightness is a public health problem all over the world, and obesity has become a common disease. Thus, Vicky et al. [52] calculated the differences in LTC costs associated with BMI in UK and found that the larger the body mass index, the higher the related costs were. Julie [53] discovered that the cost of care could be decreased after bariatric surgery in Australia. Besides, the LTC cost might be affected by the payment policies of the government, which has been proved by the work of Shota Hamada et al. [54].

Comparisons of long-term care costs under different forms of care, such as institutional care and home care, are also a focus of research in developed countries. Peter et al. [55] compared the LTC costs for high-income people with those for the farmers in institutional care and proved that the LTC cost for farmers was much less. In order to explore whether the increase in the elderly population will make long-term care costs rise, the trajectory of long-term care in 28 European countries was explored by Maria et al. [56], and it was found that the cost reduced as the expenditure of the government on health services was increased. At the same time, the elderly over 65 had turned to home care, which reflects the cost-effectiveness of home care to some extent. Leena et al. [57] studied the cost of all-day use on long-term care from 2002 to 2013 in Finland, and it was discovered that the cost in the shelters is much lower than that in nursing institutions. 

## 4. Discussion

With the acceleration of the population aging process in most countries, long-term care has gradually been accepted by the global community. After more than 20 years of research and practice, developed countries have generally recognized that the long-term care service system should be effectively separated from the medical system, and a relatively independent long-term care service system should be established. At the same time, developed countries, especially Germany, Japan, and the United States, have initially established long-term care service systems supported by public long-term care insurance system as the main body, and service standards and norms, supplemented by the active participation of family members, social workers, and volunteers. In view of the rapidly aging society in China, it is imperative to actively explore the establishment of an independent long-term care service system. However, China’s social pension and nursing function mechanism has not been paid enough attention to and developed well, especially in the sense that medical insurance does not pay long-term nursing expenses. As a result, high nursing expenses are unbearable for ordinary families. Therefore, our review systematically searched the articles on the measurement of long-term care costs from 2010 to 2019, and compared China with other countries, hoping to further expand the academic space for the study of long-term care costs in China in the future and to provide an important policy basis for the government to build a sustainable long-term care service system.

In 2010, cost accounting for home care programs was shown by a study published in the *Nursing Journal of the Chinese People’s Liberation Army* [23], and research into long-term care costs is getting more and more diversified. Until 2017 and 2018, the peak number of articles had been published so far, which means that the study of long-term care costs is gradually becoming more and more acceptable. It is acknowledged that, at the same time, academic circles at home and abroad are also quietly changing. Besides, it can be observed that qualitative research methods are applied in most studies in China, while studies around the world mainly use quantitative research methods, and the number of related articles around the world has exceeded those in China. It is believed that much related research on long-term care in various countries will appear in well-known journals at home and abroad in the future. The research objects are more extensive, and the content is more detailed. The establishment of long-term care systems in various countries is no longer empty talk.

In terms of specific research content or research perspectives, China and other countries show some similarities and differences. 

Firstly, most Chinese scholars estimated long-term care costs based on the degree of disability among the disabled elderly [24,25,26,27]. Moreover, Xu et al. used the Bayesian quantile regression method to measure the high, medium, and low levels of long-term care cost prediction for each disability state of the elderly in China from 2020 to 2050 [28], which could be a new reference for the long-term care cost system. On the other hand, there are also a large number of relevant studies based on the degree of ADL dysfunction in European and American countries [41,42]. In fact, studies on long-term care costs in European and American countries were carried out earlier. For example, Martin [58] predicted the future costs for long-term care costs in the United Kingdom, with analysis also based on the degree of disability. It can be seen that both the studies in China and in other countries estimated long-term care costs based on the degrees of disability among the disabled elderly, which implies that the accurate prediction of the scale of disabled and semi-disabled elderly will be important in the measurement of long-term care costs in China in the future.

Secondly, both China’s and other countries’ scholars are starting to pay attention to how to integrate the human value of nursing staff into the measurement system for long-term care costs, so as to achieve the goal of the scientific and reasonable measurement of long-term care costs [30,31,32]. However, the studies of European and American countries are more and more in-depth and comprehensive, and more detailed regarding the post-care cost of specific diseases, such as Parkinson’s disease [43,44,45], Alzheimer’s disease [46,47,48], colorectal cancer [49], and viral gastroenteritis [50]. 

Thirdly, the long-term care costs under different forms of care (such as institutional care, community care, and home care) have become another important topic of academic discussion regarding the development of elderly care institutions in China [36,37,38,39,40] and other countries [55,56,57]. By comparison, the relevant research in China is still a simple analysis of the comparison of long-term care costs under different forms of care, but the relevant research in other countries has begun to delve into the expectations and comparison of long-term care costs of different forms of care among patient groups with different income or education levels [55].

In conclusion, the scientific measurement of long-term care costs is an important basis for the government to formulate a long-term care policy in response to the aging population. Through the comparison between China and other countries in this review, we found that the measurement of LTC costs is more and more refined, which is mainly reflected in two aspects: the comparison of LTC costs after discharge for different chronic diseases, and the comparison of LTC costs for different nursing methods. This also provides us with the countermeasures to solve this problem in the future. Firstly, the accurate prediction of the scale of disabled and semi-disabled elderly is important for the measurement of long-term care costs in China in the future. Secondly, the human value of long-term care providers should be taken into account, and the differences of long-term care costs due for different chronic diseases should be further studied. Moreover, the comparison of long-term care costs based on different care methods (institutional care, home care, or community care) is also an important basis for policymaking. Facing increasing long-term care costs, fund-raising has become a key aspect in the construction of the long-term care service system. According to the experience of the USA, Sweden, and other countries, in the long run, public long-term care insurance (LTCI) separated from medical insurance may not be enough to fully cope with the surging long-term care costs [59]. Therefore, introducing social capital, developing private long-term care insurance (LTCI) in the private market, and realizing the effective complementarity between private long-term care insurance (LTCI) and public long-term care insurance (LTCI) may be an effective pathway [60].

At present, to cope with the increasing cost of LTC, some countries have implemented and gradually improved LTCI systems, which can be divided into two types. The first is the Nordic “welfare state” model of comprehensive public welfare, which is obviously not suitable for China’s specific national conditions. The second is the “corporatist-welfare” model, which emphasizes the equivalence of LTCI rights and obligations and is more in line with China’s current national conditions [12]. Firstly, publicly providing LTC to all of its citizens without regard to the individual need for public assistance is the basis of the LTC system in Sweden. However, it is an extremely expensive system that is fraught with the potential for abuse [19]. Secondly, home care may be cheaper than institutional care. For instance, Sweden and some OECD countries in Europe focus on providing home care versus institutional care. However, LTC public funding was almost exclusively provided in institutions due to the vast majority of LTC being provided by family and friends in USA. This is similar to in China, where most of the LTC now consists of home care and community care. In addition, how to allocate the value of LTC services, such as the manpower value of service providers, is also an important consideration in the future development of the LTC system in China.

## 5. Conclusions

This review includes 20 Chinese articles and 22 articles from other countries. Through comparison, this review draws some valuable conclusions for future academic research and policymaking. In future academic research, we should fully consider the human value of long-term care providers and further study the differences in long-term care costs due to different chronic diseases. This review was limited by the amount of current research on this topic, the search strategy utilized, the number of databases searched, and researchers’ and publication bias that may have affected the value and accessibility of the research recognized. We will continue to improve in future research.

## Figures and Tables

**Table 1:** Quality of studies.

Risk of Bias	Selection of Participants	Confounding Variables	Intervention Measurement	Blinding of Outcome Assessment	Incomplete Outcome Data	Selective Outcome Reporting
Jin et al. [23], 2010	●	△	●	●	※	●
Lu [24], 2013	●	△	△	●	●	●
Feng et al. [25], 2013	●	△	●	●	●	●
Hu [26], 2015	△	●	※	●	△	△
Yuan [27], 2018	●	△	※	●	※	●
Liu & Zhong [28], 2018	●	△	※	●	●	△
Xu et al. [29], 2019	●	●	△	●	●	●
Fengyue&Junko [30], 2018	●	※	※	●	※	●
Yu et al. [31], 2015	●	△	※	●	△	※
Fan et al. [32], 2016	●	△	※	●	●	●
Qun [33], 2013	△	△	●	●	●	●
Li et al. [34], 2018	●	△	※	●	※	●
Jin, Li, et al. [35], 2018	※	△	●	●	※	※
Yang et al. [36], 2016	●	△	●	●	●	●
Lu et al. [37], 2017	●	●	※	●	●	●
Huang et al. [38], 2012	●	△	※	●	△	●
Song et al. [39], 2016	●	△	●	●	●	●
Fen et al. [40], 2017	●	△	※	●	●	※
Ryan Greysen [41], 2017	●	△	※	●	●	△
Lagergren et al. [42], 2018	●	●	※	●	※	●
Sharada et al. [43], 2018	●	△	●	●	△	●
Larissa S et al. [44], 2012	●	△	●	●	※	●
Mitsuhiro S et al. [45], 2018	●	●	※	●	●	●
Hajime T et al. [46], 2019	※	△	△	●	※	△
Greg A et al. [47], 2015	●	※	※	●	●	●
Ramon L et al. [48], 2018	●	●	※	●	●	●
Julieta et al. [49], 2016	●	△	※	●	●	●
Saori et al. [50], 2017	●	※	●	●	●	●
Edward et al. [51], 2016	●	△	●	●	●	※
Vicky et al. [52], 2017	※	△	△	●	△	●
Julie [53], 2019	●	△	●	●	※	●
Shota Hamada et al. [54], 2019	●	△	※	●	●	●
Peter et al. [55], 2015	●	●	※	●	△	△
Maria et al. [56], 2017	△	△	△	●	●	△
Leena et al. [57], 2017	●	△	●	●	※	●

Notes: **●** Low risk of bias; **△** High risk of bias; **※** Unclear.

**Table 2:** Literature related to the measurement of Long-Term Care (LTC) costs in China.

Rank	Author, Date	Methodology	Sample	Key Conclusions
1	Jin et al. [23], 2010	Semi-structured interview	Nine general service teams of the Yin hang community health service center of Shanghai	The government should increase the price of home care services and include it into medical insurance to meet the cost compensation of home care.
2	Lu [24], 2013	Microsimulation method	The annual long-term care social insurance payment rate from 1995 to 2010	The long-term care security system makes resources flow from high-income groups to low-income groups.
3	Feng et al. [25], 2013	ADL and IADL	Community-dwelling elderly in Shanghai, 1998–2008	Demand for long-term care in Shanghai led to a surge in long-term care costs between 1998 and 2008.
4	Hu [26], 2015	Markov model	Data on disabled elderly people in China	Long-term care costs for the elderly will soar in China in the future.
5	Yuan [27], 2018	Multiple logit regression analysis	Sample survey data of disabled elders in the three provinces of the northeast	China should pay attention to the financial pressure borne by fund providers.
6	Liu and Zhong [28], 2018	Markov model	CHARLS data for 2011, 2013, and 2015	It is imperative to establish a national long-term care social assistance system.
7	Xu et al. [29], 2019	Bayesian quantile regression	The data of disabled elderly people in China from 2003 to 2016	It is predicted long-term care costs for each disability state of the elderly in China will rise sharply from 2020 to 2050
8	Fengyue and Junko [30], 2018	Field investigation	First-class nursing patients in China	Controlling labor costs and reducing material costs are the keys to reducing the LTC costs.
9	Yu et al. [31], 2015	Comparative Study	Nursing project prices in Chongqing 2014 and Guangdong 2006	It is suggested that the value of nursing technical labor services should be properly reflected when formulating the price of nursing projects.
10	Fan et al. [32], 2016	Field survey and expert consultation	A primary care patient in a tertiary hospital in Chongqing from January to March 2013	LTC costs can be included in the scope of medical insurance reimbursement.
11	Qun [33], 2013	Comparative Study	Top three elderly-hospitals in Beijing (China)	The adjustment of LTC service prices has a long way to go.
12	Li et al. [34], 2018	Essential thoughts of RBVS	2012 edition of the medical service price item specification work manual	The constructed pricing model of nursing service projects can measure the value of LTC services and fully reflect the LTC costs.
13	Jin, Li, et al. [35], 2018	Multiple linear regression	42 service items in Shanghai	Labor cost is a major factor in the cost of long-term care services.
14	Yang et al. [36], 2016	A policy evaluation of new models	51 elderly of nursing patients	The total cost of institutional care is far less than the cost of general hospital inspections and other routine expenses.
15	Lu et al. [37], 2017	Multiple linear regression	Elderly chronic patients in the urban area of Hefei	Public long-term care policies in China should focus on the distinction between institutional care and home care.
16	Huang et al. [38], 2012	Multi-status transition model	Comparison of the costing methods around the world with Taiwan, China	Through home care cost accounting, the government can provide information for the government based on the family cost data for the elderly with different functional levels.
17	Song et al. [39], 2016	Comparative study	Community and institutional care survey data	To be cost-effective, community care services should target patients with a medium physical disability.
18	Fen et al. [40], 2017	Logistic model	8500 residents aged over 60 in Jiang Ning Road were randomly selected and observed in 2014	Formal care provision in Shanghai was not determined by ADL scores but was instead more related to income.

**Table 3:** Literature related to the measurement of LTC costs in other countries.

Rank	Author, Date	Methodology	Sample	Key Conclusions
1	Ryan Greysen [41], 2017	Generalized linear model; gamma regression model; goodness-of-fit analysis	Create a nationally-representative sample of 16,673 Medicare hospitalizations for 8559 community-dwelling seniors from 2000 to 2012 using the Health and Retirement Study (HRS).	Functional impairment is associated with increased Medicare costs for post-acute care and may be an unmeasured but important marker of long-term costs that cut across conditions.
2	Lagergren et al. [42], 2018	Weighted logarithmic, linear regression	Japanese data on LTC assessments in nine Japanese municipalities, grouped. A corresponding Swedish dataset from eight varied municipalities was collected in 2002 and 2007.	The sustainability of LTC systems is a high priority in Japan and Sweden, and better decision support is needed to guide policy in this area of the welfare state.
3	Sharada et al. [43], 2018	Logistic regression; propensity score matching	Between 1994 and 2013, 7271 PD patients who met study inclusion criteria were identified in linked CPRD-HES; 7060 were matched with controls in the United Kingdom (UK).	Healthcare costs attributable to PD increase in the year following diagnosis and are higher for patients with indicators of advanced disease.
4	Larissa S et al. [44], 2012	Wilcoxon test; the generalized linear model	Claims data from the AOK Bavaria Statutory Health Insurance fund of 9147 dementia patients in 2006	Dementia poses a substantial additional burden on the German social security system, and female dementia patients need to be a key target group for health services research in an aging society.
5	Mitsuhiro S et al. [45], 2018	Multiple regression analysis	The Survey of Long-Term Care Benefit Expenditures in Japan	The societal cost of dementia in Japan appeared to be considerable. Interventions to mitigate this impact should be considered
6	Hajime T et al. [46], 2019	Cross-section analysis on time series	169 patients with Alzheimer’s disease or mild cognitive impairment in Japan	As the number of patients with Alzheimer’s disease increases, direct social costs will increase.
7	Greg A et al. [47], 2015	Polynomial regression model	3811 veterans hospitalized for ischemic stroke in Veterans Health Administration facilities in 2007	Care trajectories after stroke were associated with stroke severity and functional dependency and then had a dramatic impact on subsequent costs.
8	Ramon L et al. [48], 2018	Multiple regression model	All patients with first-ever incident ACLVI from 2002 to 2012	Hospital care costs were significantly higher than for stroke over the long term and were similar after the inclusion of the costs of institutionalization.
9	Julieta et al. [49], 2016	Kaplan–Meier method	699 patients diagnosed and treated for CRC in 2000–2006 (Spanish, Barcelona).	This study is the first to provide long-term cost estimates for CRC treatment, by stage at diagnosis and phase of care, based on data from clinical practice in Spain.
10	Saori et al. [50], 2017	Questionnaire survey	Facilities affiliated with the Kyoto Prefecture	The cost of preparing the PPE needed for the preventive measures varied among the facilities.
11	Edward et al. [51], 2016	Markov chain model	Expected prognosis of patients under alternative admission strategies over 35 years in the UK	Increasing length of stay to optimize IV thiamine replacement will place additional strain on acute care but has potential UK public sector cost savings.
12	Vicky et al. [52], 2017	Multivariable logistic regression	Adults aged over 65 in England	The increase in the need for care with BMI gives rise to additional costs in social care provision.
13	Julie [53], 2019	ABF model	All patients who had received primary bariatric surgery in a Tasmanian public hospital	At three years postoperatively versus preoperatively, episodes of care and costs reduced substantially, particularly for people with diabetes/cardiovascular disease.
14	Shota Hamada et al. [54], 2019	Generalized linear regression models	1324 residents who were admitted in 2015 in Japan	There was no apparent association between the level of long-term care needs and drug costs.
15	Peter et al. [55], 2015	Double tail test	Ontario, Canada	This analysis adds new information about the breadth of end-of-life healthcare, which consumes a large proportion of Ontario’s healthcare budget.
16	Maria et al. [56], 2017	Time-series design	The elderly aged 65 years and above of 28 European countries from 2004 to 2015	Demographic, societal, and health changes could considerably affect LTC needs and services, resulting in higher LTC related costs.
17	Leena et al. [57], 2017	Cox proportional hazard models	The elderly aged 70 years and above in 2002–2013 in Finland.	Costs of LTC decreased as sheltered housing replaced institutional LTC.
